# Work activities of primary health care nurses in Poland: National Survey Results

**DOI:** 10.1186/s12912-021-00541-2

**Published:** 2021-01-14

**Authors:** Ludmila Marcinowicz, Danuta Wojnar, Slawomir Jerzy Terlikowski

**Affiliations:** 1 Department of Obstetrics, Gynaecology and Maternity Care, Medical University of Bialystok, Szpitalna 37, 15-295 Bialystok, Poland; 2 College of Nursing, Robert Wood Johnson Foundation Executive Nurse Fellow 2012-2015, J. Bushman Endowed Chair in Nursing, Seattle University, 901 12th Avenue, Seattle, WA 98122-1090 USA; 3 Medical University of Bialystok, Szpitalna 37, 15-295 Bialystok, Poland

**Keywords:** Nurses, Primary health care, Surveys, Poland

## Abstract

**Background:**

In some countries, including Poland, nurses have acquired autonomy from being a designated “ancillary staff” to “professional staff” only in recent decades. No prior published studies have examined, however, whether the actual nursing practice in primary health care (PHC) has evolved with the advancement of education and professional autonomy. The aim of this study is to assess the scope of practice of a PHC nurses and their actual work activities.

**Methods:**

A cross-sectional study design using an investigator-developed survey was conducted in Poland, in 2018. The survey was sent to professionally active PHC nurses in Poland. Of the 225 questionnaires distributed, 202 (89.8%) were returned.

**Results:**

Out of 44 work activities examined, the most often performed activity was administering medications. Less frequent activities included recognizing patients’ nursing needs and health problems and monitoring, assessing, and interpreting basic vital signs. A correlation was found between the length of work experience and the following three activities: performing nursing care, issuing referrals for specific diagnostic tests, and ordering of specific treatments, medications, and nutritional supplements. The longer was the work experience, the more often the nurses performed nursing care (*r* = 0.15; *p* = 0.035) but less often issued referrals for diagnostic tests (*r* = − 0.24; *p* = 0.001) or orders within their scope of practice (*r* = − 0.23; *p* = 0.002).

**Conclusion:**

While nurses in general are most likely to carry out physician orders, junior nurses tend to be more likely to work toward professional autonomy and pursuing new challenges. PHC nurses in Poland perform work associated more with carrying out physicians’ orders and less with what they were prepared to do. Engaging nursing students in interprofessional education, dissemination of nursing research, and advocacy of nursing professional organizations on behalf of the profession may be an effective strategy to overcome the current barriers for PHC nurses to work the top of their license.

## Background

The need for strengthening primary health care (PHC) world-wide is at the heart of the World Health Organization’s (WHO) mission to improve population health. Over a decade ago, the WHO report entitled *Primary Health Care Now More than Ever* [1] articulated the need for a generation of new knowledge about this level of care. A more recent WHO publication is concerned specifically with the role of nurses in PHC and describes how the unique contributions of registered nurses (RNs) strengthens healthcare systems in 14 European countries [2].

Josi, Bianchi, and Brandt [3] provided evidence regarding positive contributions of RNs to overall team functioning, interprofessioanl communication, and patient centered care in primary care settings in Switzerland. Dal Molin and team found that PHC nurses significantly improved nursing-sensitive patient care outcomes, patient satisfaction, and organizational outcomes [4]. Smolowitz et al. used an exploratory descriptive design to investigate the work of PHC nurses in 16 exemplary primary care practices over time [5]. They reported that RNs across the care settings performed nine general functions including telephone triage, assessment and documentation of patient health status, chronic illness case management, hospital to community transition management, health coaching, medication reconciliation, ancillary staff supervision, and quality improvement leadership. These nursing functions significantly improved quality and efficiency of care, satisfaction of providers and other members of the healthcare team, and decreased cost of care, a finding consistent with prior reports [6, 7] and expert opinions [8].

There is substantial evidence that PCH nurses have a positive impact on chronic disease management. For example, Cruz-Cobo and Santi-Cano [9] in a systematic review of the literature provide evidence regarding the unique role of primary care nurses in improving diabetes management among insulin dependent patients. Two recently published descriptive cross-sectional investigations, one conducted in India [10] and the other in Uganda [11] demonstrate positive impact of PHC nursing education during the antenatal period on childbirth experiences and childbirth outcomes in these countries. Likewise, a recent experimental study conducted in Iraq shows positive effects of breastfeeding education provided by PHC nurses during the antenatal period on women’s breastfeeding self-efficacy during the postpartum period [12].

The role of PHC nurses has been analysed in various health care systems and perspectives, including consumers [13], nursing students [14], and nurses [15]. However, despite the consistent evidence demonstrating positive effect on patient outcomes of RN services in primary care settings, discussion regarding the optimal model of staff mix in PHC centers [16]. Aston [17] asserts that nurse generalists with specialty certification in primary care are uniquely suited to provide expanded care services to improve patient care and prevent burnout of primary care physicians and other providers in these settings, a finding consistent with prior research [6]. In summary, recent research, systematic reviews of the literature, and expert opinions, collectively lend support for broader utilization of nurses in PHC and ongoing research to better understand the work and impact of PHC nurses on patient care outcomes, work satisfaction of the PHC teams, and the cost of care.

In Poland, the PHC is the first contact for most patients within the healthcare system. The PHC system in Poland is financed by the National Health Fund. Its aim is to ensure effective and efficient care for the patient and their family, to coordinate the care, evaluate and determine care priorities, identify, eliminate, or reduce physical and mental health problems, and ensure health promotion, disease prevention, and health education activities. The patient care responsibilities are shared by the PHC team made up of a physician (the manager of care), a nurse, and a midwife. Typically, the PHC physician and nurse care-team provides care for a panel of approx. 2500 patients annually.

To offer some context to the meaning of PHC teamwork in Poland, it is important to acknowledge the cultural context in which medical and nursing professionals have practiced in recent decades. Today, nursing in Poland is an autonomous profession. Two decades ago, however, nurses were considered “auxiliary” medical personnel. The *Act on Professional Self-governance of Nurses and Midwives* passed in 1991 established a single independent professional organization to regulate nursing and midwifery care. The structure of nursing self-governance consists of the Central Council of Nurses and Midwives at the national level and Regional Chambers of Nurses and Midwives at the provincial level. The *Act on the Occupations of Nurses and Midwives* from 1996 helped to further establish the professional autonomy of nurses and midwives in Poland. Today, Polish nurses are educated consistent with the European Union (EU) requirements. Polish nurses and midwives have access to three levels of educational preparation: Bachelor of Nursing, Master of Nursing, and a PhD in Nursing Science. Additionally, postgraduate education prepares nurses for work in various specialized sectors of the healthcare system [18]. Moreover, some nurses in Poland hold specialty certifications, including PHC nurses. These nurses have completed a postgraduate qualifying course in family nursing or another field relevant to primary health care, such as pediatric nursing, community nursing, or care for chronically ill or disabled patients. A typical PHC nurse is prepared to plan and carry out a comprehensive nursing care plan within the scope of nursing practice with consideration of the point of care, patient health status, gender, level of patient abilities, and age (except for newborns and infants under 2 months of age who receive primary care exclusively from physicians). In sum, PHC nurses are well prepared to provide health education, nursing assessments and care, and coordinate diagnostic, therapeutic, and rehabilitation services.

Since January 1, 2016, when a pharmacological specialist course became available, PHC nurses are able to prescribe certain medications and dietary supplements upon completion of the course. The RNs may also issue orders for certain medical devices following physical examination of the patient. To date, no published studies have examined whether the actual use of skills and knowledge of Polish nurses in PHC is consistent with their recent expansion of educational preparation or the current scope of practice. Nurses are a large workforce in PHC and due to their scope of practice they should be considered in primary health care system staffing models [5]. Scientific evidence about nursing activities in PHC will allow the optimization and development of the practices of nurses.

This study was conducted to shed a light on the actual roles of Polish nurses in PHC and to identify potential areas for improvement. The aim of this study is to assess the scope of practice of a PHC nurses and their actual work activities.

The specific aims were to answer the following research questions:

- What work activities do PHC nurses perform the most and least often?
- Are the PHC nurses’ work activities associated with their length of work experience?

## Methods

### Study design and participants

A cross-sectional study design using an investigator-developed survey was conducted in Poland, in 2018. Convenience sampling was used, based on the availability of respondents (a real one that can be reached). The survey was sent to professionally active PHC nurses in Poland through the Regional Chambers of Nurses and Midwives who had control over identifying nurses and sending out the survey to potential respondents. Two hundred and twenty-five surveys were sent to 45 Regional Chambers of Nurses and Midwives with a request to distribute the surveys to five selected PHC nurses in each region, according to the Regional Chambers’ officials’ preferences. Of the 225 questionnaires distributed, 202 (89.8%) questionnaires were returned to the investigators.

With the exception of one respondent who self-identified as male, all of the remaining respondents were female (99.5%). The mean age of participants was 50.3 years (SD = 8.4). A large proportion of respondents was between 30 and 39 years of nursing experience (*N* = 90, 44.6%), had completed a qualification course (*N* = 193, 95.5%) and reported to work in “medium-size” towns of approximately 20,000 inhabitants (*N* = 87, 43.1%). The majority of respondents was employed on a contract bases at PHC clinics (*N* = 136, 71.8%), fewer owned or co-owned the PHC practice (*n* = 57; 28.2%) (Table 1).

**Table 1:** Sample characteristics

Characteristics	*N* = 202 (100%)
Age (years), mean, SD	Mean = 50.3 SD = 8.4
Gender	
Female	201 (99.5%)
Male	1 (0.5%)
Work experience as nurses	
0–9 years	10 (4.9%)
10–19 years	28 (13.9%)
20–29 years	49 (24.2%)
30–39 years	90 (44.6%)
40–49 years	24 (12.4%)
Qualification course	
yes	193 (95.5%)
no	6 (3.0%)
No answer	3 (1.5%)
Specialization	
yes	76 (37.6%)
no	122 (60.4%)
No answer	4 (2.0%0
Workplace location	
village	30 (14.8%)
small town	43 (21.3%)
medium-sized town	87 (43.1%)
big town	42 (20.8%)
Professional situation	
I am the owner of a medical entity or a partner in a medical company	57 (28.2%)
I have an employment contract or a contract of mandate	136 (71.8%)

Ethical approval to conduct the study was granted by the Bioethics Committee of the Medical University of Bialystok (R-I-002/290/2017). Participation in the study was voluntary and survey completion was anonymous. In the “Invitation to participate in the study” letter the respondents were assured that the provided data would be used for research purposes only and reported as aggregate data.

### Questionnaire

The survey (questionnaire) used in the study, included questions regarding the socio-demographic status of study participants and the scope of professional practice (work activities) carried out by study participants. The general format of the survey was based on the 2014 American Family Nurse Practitioner Role Delineation Study Summary Report (reference). We used some survey questions for rating work activity statements and how to analyze the data [19]. A consent to use the format was obtained from the Director of the Institute for Credentialing Research of the American Nurses Credentialing Canter. E-mail permission to use the format was issued on May 10, 2017.

A set of 44 items were prepared based on the scope of practice of a typical PHC nurse in Poland as specified in the current *Regulation* of nursing practice document [20]. The survey was developed to obtain information about the study participants’ perceptions regarding frequency of the key PHC practice activities. For example, participants were asked “How often does a primary health care nurse perform (a particular task named here) in a given year?” Possible responses included one of the following: Never; Seldom; Occasionally; Often. Participants were also asked to rate the consequences of doing the task incorrectly with possible responses: No negative consequences; Minimal negative consequences; Moderate negative consequences; Significant negative consequences. Although the question was “How often does a primary health care nurse in a given year?”, we assume that the nurses responded based on their own professional experience.

### Data analysis

Two rating scales were combined into a single measure using a hierarchical method.

Table 2 displays how the values of the overall criticality rating were constructed according to the possible survey response patterns that might be given to rate work activities by its frequency and consequence.

**Table 2:** Construction of the variable

Consequence	Frequency	Score
No negative consequences	Never	0
	Seldom	1
	Occassionally	2
	Often	3
Minimal negative consequences	Never	4
	Seldom	5
	Occassionally	6
	Often	7
Moderate negative consequences	Never	8
	Seldom	9
	Occasionally	10
	Often	11
Significant negative consequences	Never	12
	Seldom	13
	Occasionally	14
	Often	15

Statistical software IBM SPSS Statistics 20.0 was used for analysis. Descriptive statistics (frequencies and percentages, Mean, Median, Standard Deviations,) were calculated to describe the sample demographics and frequency of specific activities, and are presented to show a trend (increasing or decreasing) that describes the correlation coefficient. The calculated values of indices of particular activities were compared between the subgroups of the respondents (the length of work experience as a nurse in years: 0–9, 10–19, 20–29, 30–39, 40–49). We used nonparametric Spearman’s correlations between the indices and the length of work experience measured on the ordinal scale to assess relationships between them. Statistical significance was set at 0.05.

## Results

The most often performed activity was administering medications ordered by a doctor, including injections and medication drips (*n* = 179; 88.6%) in the PHC clinic setting. Less frequent activities included recognizing patients’ nursing needs and health problems (*n* = 176; 87.1%) and monitoring, assessing, and interpreting basic vital signs (*n* = 172; 85.1%). The list of 10 most often performed tasks reported by the participants is presented in Table 3. The least often performed tasks are reported in Table 4 and include: issuing of referrals for specific diagnostic tests (*n* = 128; 63.4%) followed by ordering specific medicinal products (*n* = 106; 52.5%) and bladder rinse (*n* = 77; 38.1%).

**Table 3:** Most often performed work activities (*n* = 202); responses “often” (N and %)

Work activity	N	%
Administering drugs ordained by the doctor, including injections and intravenous infusions	179	88.6
Recognizing patients’ nursing needs and health problems	176	87.1
Monitoring, evaluating and interpreting basic vital signs	172	85.1
Health education	166	82.2
Providing nursing care for patients with various health states and diseases	164	81.2
Diagnosis, assessment and prevention of health threats in patients	157	77.7
Providing nursing services	155	76.7
Conducting environmental interviews	151	74.8
Applying dressings to wounds, bedsores and burns	147	72.8
Collection of material for diagnostic tests	144	71.3

**Table 4:** Least often performed work activities (*n* = 202); responses “never” (N and %)

Work activity	N	%
Issuing referrals for specific diagnostic tests	128	63.4
Ordaining specific medicinal products	106	52.5
Bladder rinse	77	38.1
Performing treatments with the use of heat and cold	67	33.2
Organizing support groups	46	22.8
Performing a physical examination	42	20.8
Performing infusions or rectal ingots	41	20.3
Establishing the diet for chronically ill patients	39	19.3
Selection of feeding techniques depending on the patient’s condition	39	19.3
Bedside rehabilitation to prevent complications resulting from the disease process and prolonged immobilization	32	15.8

Short-term nurses reported less often that they were providing nursing services (Mean = 11.5; SD = 3.8; Me = 12.5) than those with the longest career (Mean = 12.9; SD = 4.2; Me = 15). However, they performed more frequently for specific diagnostic tests (Mean = 8.3; SD = 5.2; Me = 10) compared to older nurses (Mean = 3.3; SD = 4.9; Me = 0). They were also more likely to prescribe specific medicinal products (Mean = 10.4; SD = 4.2; Me = 11.5) than nurses with the most seniority (Mean = 5.6; SD = 4.0; Me = 5); (Table 5).

**Table 5:** Spearman correlations between work activities and years of work experience

Tasks	Years	Mean	SD	Me	r, *P*
Providing nursing services	0–9	11.5	3.8	12.5	*r* = 0.15
	10–19	11.1	4.3	11	0.035
	20–29	11.7	3.7	13	
	30–39	12.0	4.0	15	
	40–49	12.9	4.2	15	
Issuing referrals for specific diagnostic tests	0–9	8.3	5.2	10	*r* = 0.24
	10–19	6.5	5.6	8	0.001
	20–29	7.0	5.3	8	
	30–39	5.0	5.1	4	
	40–49	3.3	4.9	0	
Ordaining specific medicinal products	0–9	10.4	4.2	11.5	*r* = 0.23
	10–19	7.8	4.8	8.5	0.002
	20–29	8.5	4.9	9.5	
	30–39	7.0	4.5	8	
	40–49	5.6	4.0	5	

Out of 44 work activities analysed, a significant correlation was found between the length of work experience and the following three tasks: performing nursing services, issuing referrals for specific diagnostic tests, and ordering of specific medications or dietary supplements that are within the nursing scope of practice. The longer the work experience, the more often the nurses performed nursing care (*r* = 015; *p* = 0.035) but less often issued referrals for diagnostic tests (*r* = − 0.24; *p* = 0.001) or ordered specific medical products (Table 5).

## Discussion

A survey was sent to obtain information regarding the work activities Polish PHC nurses in clinical practice. The study also investigated the perceived frequency of performing the key tasks. As far as the authors of this report are aware, this is the first study exploring the PHC nurses’ scope of work in Poland. Although the most frequent activity reported by PHC nurses participating in the study was “carrying out doctor’s orders”, the other work activities included autonomous nursing activities, such as the using of nursing process to engage in patient education or health promotion activities. Study participants performed such activities as: identifying patients’ health problems and care needs, providing nursing care, conducting environmental interviews, applying dressings to wounds, bedsores and burns, and collection of material for diagnostic tests.

Observational research on nurses’ activities during home healthcare visits shows that the majority of activities were with patients with long-term illnesses and most often included: medication administration, blood samples, wound care or measurement of blood pressure; patient education was offered only in 3.5% of home visits [21].

In some countries, nurses have a long tradition of prescribing medications (I would give an example, as nurses in the US have to be advanced practice to prescribe). Research has shown that prescribing treatments or medications by nurses increases their job satisfaction [22]. In this study, however, PHC nurses, although qualified, rarely prescribed medications. An interesting finding of our study is the fact that younger nurses prescribed more frequently than more experienced nurses, who in turn were more likely to engage in basic nursing care. Bartosiewicz and Januszewicz [23], who investigated Polish PHC and clinical nurse specialists’ roles in the ambulatory care settings found a relationship between the level of burnout among the nurses and their readiness to take on new competencies such as the prescribing. Findings from their study suggest that the higher level of burnout the lower was the readiness of nurses to prescribe. In another study Zarzeka and team [24] found that while Polish nurses’ attitude toward prescribing of medications is generally positive, their perception of the right to prescribe is not, suggesting that nurses may still be in transition of taking on the new competencies.

A concerning finding of our study is the fact that PHC nurses in Poland rarely engage in organizing the support groups for patients, an activity at the heart of PHC nursing curriculum. The omission of this service may be related to many issues. It must be stressed that the number of activities performed by the PHC nurses at the point of care is very broad and ranges from arranging specialty tests to consultations and preparation for surgery, to ordering equipment for patients, administering vaccines and IV medications, to providing wound care. On the other hand, one must be mindful that PHC settings in Poland are typically managed by general practice physicians who employ nurses, and therefore, control their nursing scope of practice regardless of the nurses’ educational preparation. This may have contributed, at least in part, to the fact that a few nurses reported prescribing medications or facilitating patient support groups. Expanding these activities by PHC nurses in the future could enhance patient outcomes and satisfaction and possibly job satisfaction as nurses are encouraged to practice at the top of their license [25].

Qualitative research on primary care nursing activities with patients affected by physical chronic disease and common mental disorders, shows that there is a gap between the types of activities that are expected of primary care nurses and what they actually do. The authors of the study conclude that the potential of PHC nurses is not always fully exploited [26].

In contrast to other developed countries, such as the United States, where nursing’s scope of practice has long been established and regulated by the nursing profession [19], findings from our study can be viewed as a starting point in understanding the patterns of Polish nurses’ work in PHC. The qualitative research among Polish patients shows that although the traditional model of nursing care and the hierarchical relationship with the dominant position of the doctor are emphasized in the statements of patients, the perception of the nurse as a source of information, both for the doctor and the patient, is also emphasized [27].

The importance of PHC nurses in providing community care, from interpersonal to inter-organizational and system level, is confirmed by the scoping review of the current literature from various countries. The authors of the review emphasize that the awareness of the scope of nurses’ practice by the government and health authorities as well as primary health care allows nurses to practice to the full extent [28].

Primary care services are constantly evolving in response to the needs, available resources, health policies, as well as the expectations and needs of consumers in a given country. The results of our research provide new scientific evidence on the professional activity of primary care nurses in the context of national specificity. They can be used in the ongoing discussion and debate on the role and scope of nurses’ services in the primary health care system and set directions for the professional development of this group of service providers. Moreover, they can be used in updating the nursing education system, especially postgraduate education.

Other researchers, through their research, have the chance to identify problems in their own country and demonstrate international differences through the exchange of experiences.

This study has several limitations: first, the Regional Chambers of Nurses and Midwives were free to choose which PHC nurses would receive the questionnaire, which could have led to selection bias. Because we used a convenience sampling, the sample may not represent the population. Second, the proportion of participating nurses with a few years of nursing experience was significantly lower than nurses with many years of experience, which limits the generalizability of findings. More study participants with fewer years of professional experience might have produced different findings. When interpreting the results of our research, we should also take into account the lack of men in our sample (only one man). Moreover, the survey instrument may not have included all nurse activities in PHC. Qualitative research shows that nurses also perform tasks not related to nursing [29].

## Conclusions

While nurses in general are most likely to carry out tasks on the orders of a physician, junior nurses tend to be active in working on professional autonomy and new challenges.

PHC nurses in Poland perform work associated more with carrying out physicians’ orders than the nursing work for which they were prepared. Engaging nursing and medical students in interprofessional education, attendance at conferences and dissemination of nursing research through conference presentation and publication, and advocacy of nursing professional organizations on behalf of the profession may be an effective strategy to overcome the current barriers to practicing PHC nursing at the top of the license. The findings of our study may also be used to update the content of qualification and specialist courses for PHC nurses. Research is needed to identify potential differences in how PHC nurses with fewer years of professional experience carry out their work activities. Moreover, repeating the study in a few years with a larger and more diverse sample to identify any generational differences and potential changes in Polish PHC nurses’ work to better align with that of PHC nurses in other developed countries is needed.

## Data Availability

The datasets used and/or analysed during the current study are available from the corresponding author on reasonable request.

## Abbreviations

PHC: Primary Health Care
WHO: World Health Organization
RN: Registered Nurse
